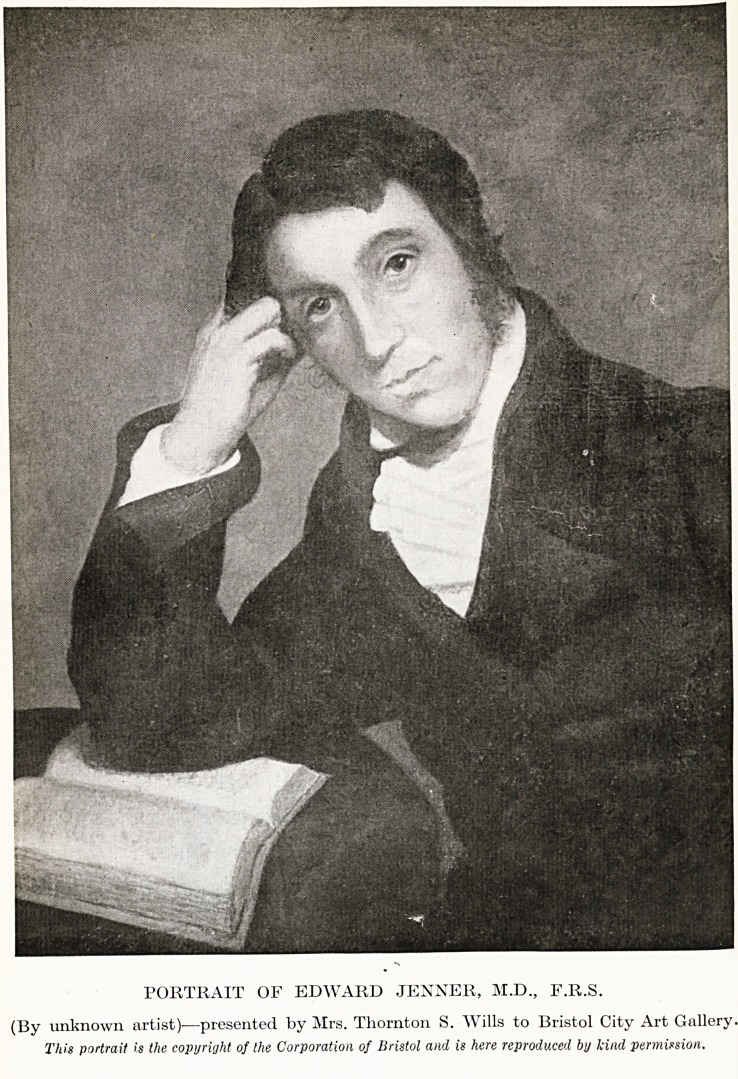# Edward Jenner

**Published:** 1948

**Authors:** T. Milling


PORTRAIT OF EDWARD JENNER, M.D., F.R.S.

(By unknown artist)—presented by Mrs. Thornton S. Wills to Bristol City Art Gallery.

*This portrait is the copyright of the Corporation of Bristol and is here reproduced by kind permission.*

[*Frontispiece*

### The Bristol Medico-Chirurgical Journal

*"Scire est nescire, nisi id me
Scire alius sciret."*

SPRING, 1948.

### EDWARD JENNER

The Presidential Address, delivered on Wednesday, 8th October, 1947, at the opening of the Sixty-ninth Session of the Bristol Medico-Chirurgical Society.

BY

T. Milling, M.B., Ch.B.

Jenner was born in 1749 in the vicarage, being the third son of the Rev. Stephen Jenner, vicar of Berkeley. His father died when he was five years old and Edward was brought up by his elder brother Stephen, who succeeded his father as vicar. Jenner's mother was "the daughter of Rev. Henry Head of an ancient and respectable family in Berkshire." He formerly held the living of Berkeley and was a prebendary of Bristol Cathedral.

The old vicarage is no longer in existence. Jenner bought the house known as "The Chantry," and it was here he lived and died. It is now the vicarage. In the grounds, there still stands the little hut where Jenner used to vaccinate the children of the neighbourhood gratuitously. He called it the Temple of Vaccina. Many of the trees are old enough to have been planted by him and the room where he died is still pointed out.

Jenner went to school at Wotton-under-Edge and later at Cirencester. We are told that he early evinced an interest in geology and natural history. At thirteen, he was sent as an apprentice to Daniel Ludlow, a surgeon living at Sodbury. He stayed with him for six years and then went, as a house pupil, to the great surgeon and pioneer John Hunter in London. His name is entered as a student at St. George's Hospital in 1770.

It was singularly fortunate for Jenner that he should meet John

Hunter. They were mutually attracted. Hunter was his senior by twenty-one years, but the common bond of interest in biology cemented a friendship which lasted as long as Hunter lived.

They kept up a life-long correspondence and many are the strange requests for natural history specimens which Hunter, in his rather crude English, made from Jenner when he had settled in a country practice.

Jenner did his work so well and impressed his master so much with his ability that, when Captain Cook returned from his first voyage to the Southern Hemisphere, Hunter recommended Jenner for the job of arranging and preparing the mass of biological material which had been collected on the voyage. This, in turn, led to his being offered the post of biologist to Cook's second voyage. This tempting offer was declined by Jenner. Hunter's marked appreciation of Jenner's attainments was again shown when he formed the plan of a school of natural history and human and comparative anatomy—the first of its kind in this country—and asked him to come and be his partner in the undertaking. Whatever may have been the reason, after two years as Hunter's pupil he determined to return to the simple, if strenuous life of a country doctor in Berkeley. He loved the countryside and Berkeley was specially attractive to him—the trees, the birds, the flowers, the opportunities to satisfy his interest in geology, but more particularly the warm-hearted country folk with whom he felt so much at home.

In the treatment of many diseases, his views, founded on the improved anatomy and physiology he had learned from Hunter and his own acute observation, were far in advance of his time. But it was chiefly by his sympathetic qualities of heart that Jenner most of all obtained and maintained the influence he possessed. He is described by his friend Edward Gardner, who had been a school-fellow of Chatterton, about this time as follows :—" His height was rather under the middle size, his person was robust, but active and well formed. In his dress he was particularly neat, and everything about him shewed the man intent and serious, and well prepared to meet the duties of his calling. When I first saw him, it was on Frampton Green. I was somewhat his junior in years and had heard so much of Mr. Jenner of Berkeley that I had no small curiosity to see him. He was dressed in a blue coat and yellow buttons, buck-skins, well-polished jockey boots, with handsome silver spurs, and he carried a smart whip with a silver handle. His hair, after the fashion of the times, was done up in a club and he wore a broad-brimmed hat. We were introduced on that occasion. . . ." Sir D'Arcy Power adds that he usually rode a white horse and altogether made an attractive, smart and cheery figure.

During this part of his career when he was actively engaged in his practice he found time to conduct many biological experiments, some of which were suggested by Hunter. In fact, Hunter rarely wrote without some reference to this work. Jenner had occasion to send Hunter patients from time to time for his opinion and the report on these is accompanied by requests for biological material or suggestions of experiments which might help to solve some biological problem. He studied the subjects of the torpidity of hibernating animals and migration of birds : he introduced an improved method of purifying tartar emetic and conducted experiments on the effect of animal manure on vegetation. He shewed "it was first the robin, not the lark, as has been generally imagined, as soon as twilight has drawn the imperceptible line between night and day, who begins his lonely song."

Jenner anticipated Darwin in his observations on the earthworm —he said that the earthworms, particularly about the time of the vernal equinox, were much under and along the surface of our moist meadowland and wherever they move they leave a train of mucus which becomes manure to the plants. In this respect, they act as the slug does in furnishing material for food for the vegetable kingdom, and under the surface they break the stiff clods in pieces and finally aerate the soil.

Jenner managed to get a whale for Hunter and had it sent to London. In the days of stage-coaches this would have been impossible by land and its condition would have been rather fruity by the time it reached London, so I presume it was sent by sea in a coastal trader.

His original researches into the habits of the cuckoo were presented by Hunter, on his behalf, to the Royal Society and though they were not generally accepted by naturalists at the time, their accuracy has since been amply confirmed. He shewed that the cuckoo laid many eggs during her short stay in this country, sometimes in the nest of one bird and sometimes in that of another, the egg of the host being imitated in each case except that of the hedgesparrow. The hedgesparrow is apparently not at all disturbed at finding an odd egg in its nest and hatches all alike. It was known that the young cuckoo alone survived in the nest of its host and it was thought that the adult cuckoo removed the eggs of the host, but it was Jenner who proved that was not so. He shewed that the young cuckoo, from two–twelve days old, by means of a peculiar formation of its back, was able to hoist the hedgesparrow's eggs out of the nest, using its wing-tips to lever itself up to the top of the nest : or, in the event of the hedgesparrow's eggs being hatched, the more-rapidly-growing young cuckoo contrived to get the babies on to its back and heave them out of the nest. Photography has proved Jenner to be correct in this observation. He was interested, too, in the times of arrival and departure of the adult cuckoos and the time of the first emigration of the young cuckoo.

It was shortly after this paper on the cuckoo that Jenner was elected a Fellow of the Royal Society. " It has ever been looked on," says Baron, referring to the paper, " as a specimen of accurate and successful investigation . . . affording to every subsequent naturalist a plain, convincing and instructive account of a subject which up to that time had been involved in the greatest obscurity." This investigation of the habits of the cuckoo is Jenner's chief claim to fame as a naturalist, but just before his death he was preparing a paper for the Royal Society on the migration of birds, which was published by Rev. C. C. Jenner in the November following Jenner's death.

In 1778 he had an unhappy love affair. The lady's name is not mentioned, but apparently she turned poor Jenner down, and as he was a man of deep feeling and deadly in earnest he was correspondingly depressed and wounded in spirit at her refusal.

It was ten years later that he married Catherine Kingscote, a lady of one of the most ancient and respectable families in Gloucester. She was always a delicate woman, but devoutly religious, and Jenner found in her counsel and sympathy solace in many of the most trying scenes of his future life. His eldest son, Edward, was born on the 24th of January, 1789. John Hunter was his godfather.

He had three children : Edward, born 1789, died 1810 ; Catherine, married 1822, died August 5th, 1833 ; Robert Fitzharding Jenner survived his father.

His domestic life was very happy and the peace and quiet of Berkeley suited him well and he had many and influential friends, including the scion of the house of Berkeley. He was a cheerful and sociable fellow, says Hale White, popular with his fellow doctors and with his patients. He frequently spent days in the house of particular friends, especially if any of them were ill, carrying on his practice from his temporary headquarters.

Educated people loved his conversation and he used to encourage those whom he liked to ride with him on his rounds. When he left a patient's house, often some of the family would ask permission to ride home with him even if it was midnight. His range of conversation was vast ; he was often witty, was fond of epigram and was no mean poet ; also he could sing and play the violin and flute.

He liked to form parties for outings, and Barrow Hill was one of his favourite haunts, from which place there is a magnificent view of the Forest of Dean and Bristol Channel. He particularly liked to watch the sunset from this spot. I had great difficulty in locating it, but the journey was well worth while and I can confirm Jenner's opinion of its beauty—the great sweep of the Severn which almost encircles the hill from Sharpness with its docks on the left, round past Newnham and Westbury, on the opposite bank : and still further to the right, I could see, on the near bank, an outcrop of geological strata (similar to that at Aust) where Jenner used to collect fossils.

Now all his study of biological problems was closely allied to his work as a medical man and was carried out in conjunction with his daily work as a doctor. In fact, his approach to medicine was that of a scientist always enquiring into the why and the wherefore of things. He used to examine the bodies of animals slaughtered for food or which had died of disease, and he took every opportunity to perform post-mortem examinations on those of his patients who died. His conclusions were not always correct—subsequent knowledge has proved him to have been wrong. For instance, he believed that tubercles in the lungs were but a stage in the development of hydatid cysts.

But he made some remarkably good shots. He was the first man ever to describe disease of the coronary arteries and to associate it with anginal pain. He communicated his observations to the Medico-convivial Society which met at Rodborough. This paper and others fell into the hands of some member of the Society and he could never recover them.

Fortunately, his claim to priority in the discovery of this disease is substantiated by his old schoolfellow, Dr. Parry of Bath, who, in his "Inquiry into the Symptoms and causes of Syncope Anginosa," says: "To some questions which I have lately put to that excellent pathologist (Jenner), as to the series of the observations which produced that opinion, I have received the following answer:

"The first case I ever saw of angina pectoris was that in the year 1772, published by Dr. Heberden, with Mr. Hunter's dissection. There, I can almost positively say, the coronary arteries of the heart were not examined. Another case of a Mr. Carter, at Dursley, fell under my care. In that, after having examined the more important parts of the heart without finding anything by means of which I could account either for his sudden death or the symptoms preceding it, I was making a transverse section of the heart pretty near its base when my knife struck against something so hard and gritty as to notch it. I well remember looking up to the ceiling, which was old and crumbling, conceiving that some plaster had fallen down. But, on a further scrutiny, the real cause appeared; the coronary arteries were become bony canals. Then I began a little to suspect. Soon afterwards, Mr. Paytherus met with a case. Previously to our examination of the body, I offered him a wager that we should find the coronary arteries ossified. This, however, proved not to be exactly true; but the coats of the arteries were hard, and a sort of cartilaginous canal was formed within the cavity of each artery, and there attached, so, however, as to be separable as easily as the finger from a tight glove. We then concluded that malorganisation of these vessels was the cause of the disease.

"At this very time my valued friend, Mr. John Hunter, began to have symptoms of angina pectoris strongly marked upon him; and this circumstance prevented any publication of my ideas, as it must have brought on an unpleasant conference between Mr. Hunter and me. I mentioned both to Mr. Cline and Mr. Home my notions of the matter at one of Mr. Hunter's Sunday-night meetings, but they did not seem to think much of them. When, however, Mr. Hunter died, Mr. Home very candidly wrote to me immediately after the dissection to tell me I was right.

" The appearance in Mr. Bellamy's case gave me the idea that the disease arose from a determination to the vasa vasorum and that the concretions were deposits from the coagulable lymph or other fluids which had oozed out on the internal surface of the artery."

Jenner also drew attention to disease of the heart associated with rheumatic fever and was thus a pioneer in this field of pathology. He was at least suspicious that pulmonary tuberculosis was an infectious disease and was, in this respect, fifty years in advance of general medical knowledge at that time. But, of course, it was his discovery of vaccination which made him famous all over the world as the greatest benefactor, till then, of the human race, and famous for all time as the pioneer of preventive inoculation against disease. Out of Jenner's work has grown the science of Bacteriology and Pasteur, born a month before Jenner's death, most generously declared that to Jenner should be given the credit of the discovery of the attenuated virus. It was at Pasteur's request that the name " vaccine " instead of some new name was given generally to matter introduced in preventive inoculation even though the cow has now nothing to do with it. " I have ", said Pasteur, " given to the term vaccination an extension which Science, I hope, will accept as a just homage to the immense service rendered by one of the greatest of Englishmen—Edward Jenner. How great to me is the happiness to be able to honour his immortal name in this noble and hospitable city of London."

Jenner was interested in the subject of Cowpox from the earliest days of his medical career. When the subject of smallpox was mentioned at Dr. Ludlow's in Sodbury, he heard a woman say that she could not take that disease because she had had cowpox. He mentioned this local tradition to John Hunter when he went to London, but that worthy man apparently put little on it though he quoted it in his lectures to students.

Jenner's attention was further drawn to the subject when he began general practice in Berkeley by observing that in performing the customary inoculation with variolous material, some people did not take the infection and that these were the very people who had had cowpox. He used to discuss the subject with his colleagues in the medical societies at Rodborough and Alveston. They, of course, were well aware of the local tradition but considered it little more than the gossip of old women. They said that they knew of many cases of smallpox occurring in those who had had cowpox. Jenner knew this to be true, but he did not let the matter rest there. He wanted to know why it should be so. He discovered first that what was referred to as cowpox by the local inhabitants was not always one and the same thing—other vesicles and ulcerations occurred on the cows' udders besides true cowpox and all alike were called cowpox. He learnt by careful observation to distinguish the true from the spurious cowpox. But, for his discomfiture, he discovered that even those who were infected with true cowpox sometimes developed smallpox subsequently and his next discovery was that it was only at a certain stage of vesicle that protection could be acquired. When the vesicle has become purulent only septic organisms are transmitted and though a vesicle may develop, the person is not protected from smallpox.

These investigations took a long time but he was now in a position to put the knowledge which he had acquired to the test of experiment. He says himself: ". . . During the investigation of the casual cowpox, I was struck with the idea that it might be practical to propagate the disease by inoculation after the manner of smallpox, first from the cow, and finally from one human being to another. I anxiously waited some time for an opportunity of putting this theory to the test. At length the period arrived, and the first experiment was made upon a lad of the name of Phipps, in whose arm a little vaccine virus was inserted, taken from the hand of a young woman (Sarah Nelmes) who had accidentally been infected by a cow. Notwithstanding the resemblance which the pustule thus excited on the boy's arm bore to variolous inoculation, yet as the indisposition attending it was barely perceptible I could scarcely persuade myself the patient was secure from smallpox. However, on his being inoculated some months afterwards, it was proved that he was secure. This case inspired me with confidence ; and as soon as I could again furnish myself with virus from the cow, I made an arrangement for a series of inoculations. A number of children were inoculated in succession, one from the other ; after several months had elapsed, they were exposed to the infection of smallpox —some by inoculation, others by variolous effluvia, and some in both ways, but they all resisted it. The result of these trials gradually led me into a wider field of experiment, which I went over not only with great attention, but with painful solicitude."

Jenner believed that cowpox and smallpox were one and the same disease, that cowpox was derived from a disease affecting the heels of horses, known as "the grease" ; which, whether casually or by vaccination, gave the same protection from smallpox as either an attack of smallpox or inoculation with smallpox material—no more and no less ; when, as occasionally happened, a person who had been vaccinated subsequently developed smallpox, he pointed out that the same applied to inoculation with variolous material and that second attacks of smallpox were not unknown—he did not recognize that the beneficial effects of vaccination began to wear off in time and that there was need for re-vaccination. He believed it was possible to pass the protective cowpox from arm to arm and so to spread its virtues that smallpox might be abolished from all the world by universal adoption of vaccination—he was not aware of the possibility of passing on other diseases at the same time, the realization of which caused the decline of arm-to-arm vaccination.

The remarkable perseverence with which Jenner pursued this subject and the interest he sustained in it all his life constitute one of the most notable things about him. He had indeed followed Hunter's advice: "Don't think, try; be patient, be accurate." The accuracy of his work, and, in the main, the conclusions he drew from it, have stood the test of time.

I do not intend to go into the story of his struggle to disseminate the knowledge of vaccination and its beneficial effects: of how he impoverished himself by neglecting his practice, by frequent visits to London to instruct others in the technique of vaccination, to correct the errors of his early adherents and to combat those who opposed it on principle. All this and much more of his story may be found in Baron's life and in the centenary number of the *B.M.J.*, 1896, and in Hale White's address at the centenary of his death in 1923, before the Royal Society of Medicine.

The last twenty-five years of his life were devoted to pressing home his success, and inspired by the vision of the eradication of smallpox, he devoted himself to spreading the good news. His son Edward's death from pulmonary tuberculosis in 1810 was a sad blow. But a greater blow was the death in 1815 of his wife. The preceding five years he spent with her at Cheltenham. He tended her with loving care, and after her death, stricken with grief, he retired finally to end his days at Berkeley. He was in poor health and was already showing signs of impending dissolution. In 1820 he had a seizure from which, however, he recovered; but he remained ever after sensitive to high-pitched sounds. He says of himself: "I boast of my strength in the morning, but evening comes too soon. Such are the workings of the old partnership of mind and body when the firm has been long established."

He had been made a J.P., and the post-war unemployment and distress kept him fully occupied. The day before his death, he had walked to the neighbouring village of Ham to distribute food to the poor and was apparently in good health. The following morning, however, he was found unconscious on the floor of his study and died the following day of cerebral haemorrhage. Baron, his friend and biographer, attended him in his final illness.

His book, called *An Inquiry into the causes and effect of Variolae Vaccinia, a disease discovered in some of the Western counties of England, particularly Gloucestershire, and known by the name of Cowpox,* consists of only seventy-five pages and is a pattern, even to-day, of what a report on a piece of research should be. He insisted on the details of his method, neglect of which led to some of the early mistakes—London doctors were not willing to be taught by a country doctor. No Englishman, in private life, has ever attained the world-wide renown that Jenner did. The news of vaccination spread with miraculous rapidity, when one considers the means of transport and communication available in those days, and it was taken up with enthusiasm in Vienna and later in Italy, Germany, France and America. The anniversary of the date of the first vaccination, May 14th, was held as an annual festival in Berlin for many years afterwards; they raised a temple as a memorial to him at Brunn in Austria, Napoleon had a medal struck in his honour in 1804, and at Jenner's request, released several Englishmen detained in France, caught up by the declaration of war.

He received the freedom of many cities and honours and congratulations from many countries. A list of his honours and awards is to be found at the end of Baron's life. Perhaps the most notable of these is that he was made an Associate of the Imperial Institute of France in 1811 and Hon. M.D. of Oxford University in 1813—a distinction very rarely bestowed. But, with all these honours and his association with distinguished people—the Prince of Wales, the Empress of Russia, the King of Prussia—he remained unspoiled, "the singleness of his heart and his genuine modesty graced and adorned his splendid reputation." Such was the man to whom the world was indebted for vaccination; no court or metropolitan physician, no university student, but a country doctor, a man of science and of benevolence whose name is undying.

---

I wish to acknowledge the help and advice, in the preparation of this address, of Dr. Ashworth Underwood, Wellcome Historical Medical Museum; Mr. A. E. S. Roberts, Bristol Medico-Chirurgical Society Library.

### REFERENCES

Baron.—*Life of Edward Jenner.*

*B.M.J.*—May 23rd, 1896; Feb. 3rd, 1923.

Sir William Hale White.—*Great Doctors of the 18th Century.*

F. Dawtrey Drewitt.—*Life of Edward Jenner.*

Gumston.—*History of Medicine.*

Sir D'Arcy Power.—*British Masters of Medicine.*

G. T. Bettany.—*Eminent Doctors.*

H. G. Brown.—*Bristol, England.*

Green's *History of the English People.*

E. Wynn Williams.—*The Kingsway Histories.*

## Figures and Tables

**Figure f1:**